# CIP2A silencing alleviates doxorubicin resistance in MCF7/ADR cells through activating PP2A and autophagy

**DOI:** 10.1007/s12094-021-02616-7

**Published:** 2021-05-04

**Authors:** Z. Zhu, Z. Wei

**Affiliations:** 1 Department of Radiotherapy, Cangzhou Central Hospital, No.16 Xinhua West Rd, Cangzhou city, Hebei Province 061000 China; 2 Thyroid and Breast Department, Cangzhou Central Hospital, No.16 Xinhua West Rd, Cangzhou city, Hebei Province 061000 China

**Keywords:** CIP2A, MCF7 cell, Doxorubicin, PP2A, Autophagy

## Abstract

**Background:**

Cancerous inhibitor of protein phosphatase 2A (CIP2A) plays a critical role in the pathogenesis of various types of cancer. Here, we investigated whether manipulating CIP2A abundance could enhance the treatment effects of doxorubicin in MCF-7/ADR cells.

**Methods:**

*CIP2A* silencing was achieved by specific siRNAs. Proliferation of breast cancer cell line MCF-7/ADR under effective doxorubicin concentrations after *CIP2A* silencing was examined by MTT assay. Wound healing assay was performed to quantify cell migration and caspase-3/-7 activities were measured for assessing the extent of apoptosis.

**Results:**

First, our data confirmed that MCF-7/ADR cell proliferation was suppressed by doxorubicin in a dose-dependent manner. Additionally, knocking down of *CIP2A* could further decrease MCF-7 cell proliferation and migration, even in the presence of doxorubicin. Mechanistically, we have found that *CIP2A* silencing promoted cell apoptosis relative to doxorubicin alone or vehicle control groups. Lastly, phosphatase2A (PP2A) activity was potentiated and the autophagy markers, LC3B and Beclin1, were upregulated after knocking down *CIP2A*.

**Conclusion:**

Our findings support the potential benefits of using CIP2A inhibitor as a therapeutic agent to treat doxorubicin-resistant breast cancer.

**Supplementary Information:**

The online version contains supplementary material available at 10.1007/s12094-021-02616-7.

## Background

Protein phosphatase 2A (PP2A)—an enzyme that regulates cell cycle and apoptosis [1, 2]—is a well-established tumor suppressor whose inhibition leads to neoplastic transformation [3]. Cancerous inhibitor of PP2A (CIP2A) was involved in the development and pathogenesis of many cancer types [4, 5]. CIP2A was first identified as a novel partner for PP2A by tandem mass spectrometric analysis and then designated as a cellular inhibitor of PP2A due to its ability to interact with the oncogenic transcription factor c-Myc, and prevent it from PP2A mediated proteolytic degradation [6]. CIP2A overexpression induces transformation in human cells. In contrast, its RNA interference (RNAi)-mediated knockdown of CIP2A leads to tumor regression in xenograft mouse models [7–9]. These studies also suggested that CIP2A plays a critical role in tumorigenesis via preventing c-MYC protein degradation [10].

Doxorubicin is the most effective anthracycline antibiotics and widely used as a chemotherapy medication to treat various types of cancer, including breast cancer [11]. Mechanistically, cancer cell death waste induced by topoisomerase II-α (TOP2A) poisoning under doxorubicin treatment [12]. Although breast cancer is the most sensitive solid tumors to chemotherapy, the initial responsive cancer cells frequently developed resistance to cytotoxic drugs, including doxorubicin. Nearly 50% of breast cancer patients had failed treatment due to growing resistance to doxorubicin [13]. The most common mechanisms of resistance include reduced intracellular drug concentrations, increased drug metabolism enzymes, and deregulation of cellular apoptotic pathways. Recent reports indicated that lack of functional p53 has a contribution to doxorubicin resistance due to reduced apoptotic activity [14].

Recent studies reported that CIP2A was closely linked to aggressive potential and sensitivity to doxorubicin treatments in breast cancer cells [9, 15]. Several studies revealed that CIP2A was involved in chemoresistance via controlling cell growth and autophagy [16, 17]. To determine whether manipulating CIP2A levels could have any influence on the treatment effects of doxorubicin, we tested the biochemistry characteristics of MCF-7/ADR cells under effective doxorubicin concentrations via RNAi-mediated *CIP2A* silencing. Furthermore, PP2A activity and cell autophagy were detected with the aim of unveiling the possible underlying functions of CIP2A.

## Materials and methods

### Cell cultures

MCF-7/ADR cells were cultured in Dullbecco’s Modified Eagle’s Medium (DMEM, Gibco) with 10% fetal bovine serum (FBS, Gibco). To determine the effective concentration of doxorubicin, MCF-7/ADR cell was cultured with a serial of concentrations of doxorubicin (20 μM, 40 μM and 80 μM).

### Proliferation assay

MCF-7/ADR cells were washed three times with phosphate-buffered saline (PBS) and detached in 0.25% trypsine–EDTA. Then, cells were seeded into 96-well plates in total 100 μL of complete culture medium per well. After 24 h, 15 μL of MTT reagent and 100 μL 20% sodium dodecyl sulfate (SDS) were added to each well for 4 h. The optical density (OD) was recorded on a plate reader using 570 nm and 630 nm wavelengths.

### siRNA and validation experiments

The CIP2A siRNAs were from GenePharma company (Shanghai, China). *CIP2A* siRNA1 sequence is 5′-CUGUGGUUGUGUUUGCACUTT-3′; siRNA 2 is 5′-ACCAUUGAUAUCCUUAGAATT-3′. As a negative control, a scrambled siRNA was used. When MCF-7/ADR cells reached 30–50% confluence, 50 nM CIP2A or scrambled siRNA with Lipofectamine 2000 reagent was mixed for 5 min at room temperature and added into cells. To determine knockdown efficiency, transfected cells were collected and lyses for 30 min on ice with radio-immuno-precipitation (RIPA) buffer. Then, equal amounts of protein were subjected to immunoblotting.

### Wound healing (scratch) assay

After MCF-7/ADR cells were cultured for 24 h in six-well plates, a plastic tip was used to scratch confluent cells. The images of closed area were recorded with inverted microscopy at different time courses. The distance of the open area was quantified by averaging the length of 6–8 randomly drawn horizontal lines.

### Analysis of caspase activation

To assess cell apoptotic rate, we utilized Caspase-Glo^®^ 3/7 Assay kit (Promega) for their value test. All steps were performed according to manufacturer’s instructions. The activities of caspase-3/7 were recorded following luminescence reading.

### PP2A activity assay

Phosphatase assay of cells was used for PP2A activity test, which was detected using a PP2A assay kit (Millipore Sigma, USA). Absorbance value at 660 nm was recorded in spectrophotometer. The values of PP2A activity in different groups were normalized with control group.

### Immunoblot analysis

Cells were incubated with RIPA buffer for 30 min on ice. Equal amounts of total protein were loaded into 10% SDS–polyacrylamide (SDS-PAGE) gel for electrophoresis, and then transferred onto Immobilon-NC membrane. The membranes were blocked with 5% non-fat milk for 60 min. After three times of washing steps, membranes were incubated with antibodies against LC3B, Beclin 1and β-actin for 24 h at 4 °C and secondary antibodies for one hour at room temperature. The blots were visualized with chemiluminescence (Amersham Bioscience). Densitometrical measurement of the bands of interest was analyzed using the ImageJ software.

### Autophagy flux assay

mRFP-GFP-LC3 tandem fluorescent protein was used to measure autophagy flux. GFP is quenched in acidic environments, while mRFP is much stable and fluorescences even at acidic pH found in lysosomes. Therefore, the formation of autophagosome leads to an increase of yellow puncta, while autolysosome formation leads to increased red puncta. Briefly, cells were infected with mGFP-RFP-LC3 adenovirus (Hanbio) for 2 h and changed to complete medium. Confocal fluorescent microscopy (Zeiss LSM980) was used to observe the LC3 puncta 24 h after transduction.

### Statistical analysis

SPSS software (V17.0) was used to analyze data with the Student's t test or one-way analysis of variance (ANOVA) methods. The means ± SD was shown for all experiments (*N* = 3). *P* values equal to or less than 0.05 were considered statistically significant.

## Results

### Proliferation of MCF-7/ADR cells with doxorubicin treatment

To determine the effective concentration of doxorubicin that affects proliferation of breast cancer cell lines, MTT assay was used in MCF7 cell treated with various concentrations of doxorubicin. We have found that 40 μM and 80 μM doxorubicin caused a significant decline in terms of the growth rate of MCF-7/ADR cells, while 20 μM doxorubicin had no dramatic effect (Supplementary Fig. 1). These results indicated that doxorubicin inhibited MCF-7/ADR cell proliferation in a dose-dependent manner.

### Proliferation of MCF-7/ADR cells under doxorubicin treatment after CIP2A silencing

**Fig. 1:**
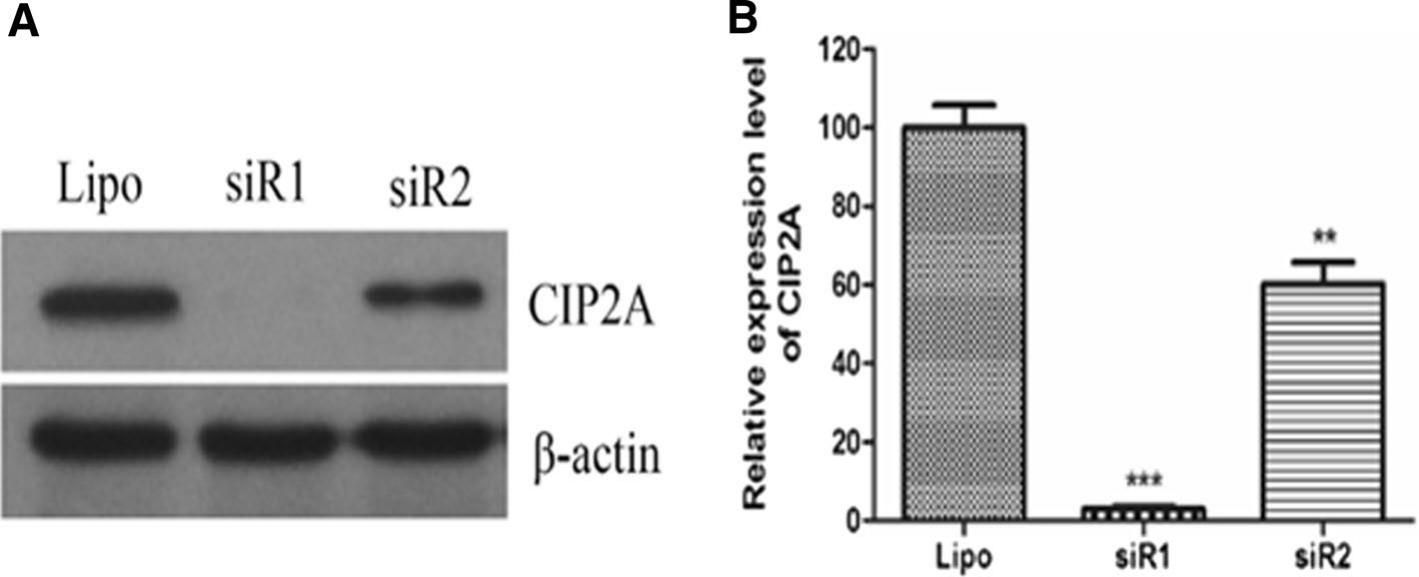
Detection of efficiency of CIP2A RNA interference. **a** Western blotting assay. **b** Quantification of the western blotting bands. **P < 0.01, ***P < 0.001 compared to Lipo control. *Lipo* lipofectamine 2000

To investigate role of CIP2A on breast cancer cell growth, we performed *CIP2A* gene silencing experiment and found that *CIP2A* levels were indeed downregulated with two different siRNA sequences (siR1 and siR2), leading to complete and partial knocking down results, respectively (Fig. 1a, b).

**Fig. 2:**
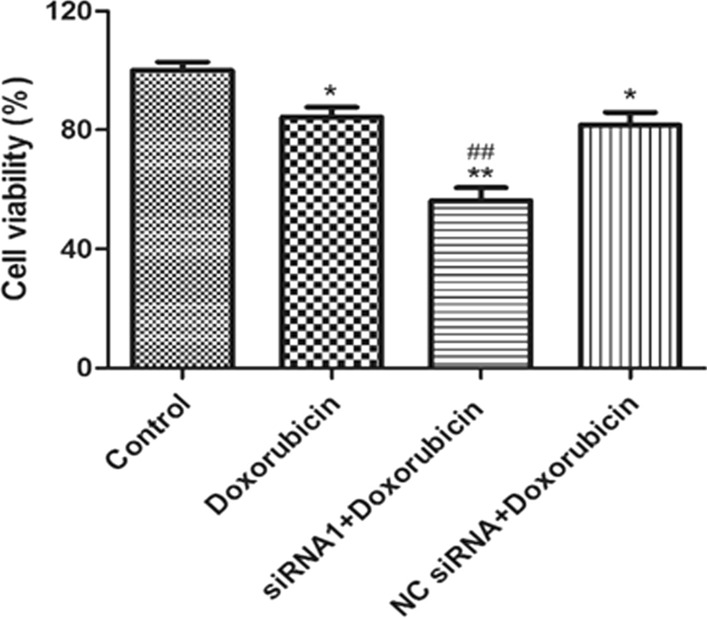
Detection of cell proliferation by MTT assay. *P < 0.05, **P < 0.01 as control, ^##^*P* < 0.01 compared to Doxorubicin-treated group

Consistent with our finding shown in Fig. 1, doxorubicin (40 μM) alone significantly inhibited MCF-7/ADR cell proliferation compared to vehicle. Interestingly, knocking down of *CIP2A* leads to a dramatic decrease of cell viability on top of doxorubicin-mediated reduction (Fig. 2). These results suggested that *CIP2A* silencing could promote cell death in cell lines that have developed resistance to doxorubicin.

### CIP2A silencing suppresses cell migration and promotes cell apoptosis

**Fig. 3:**
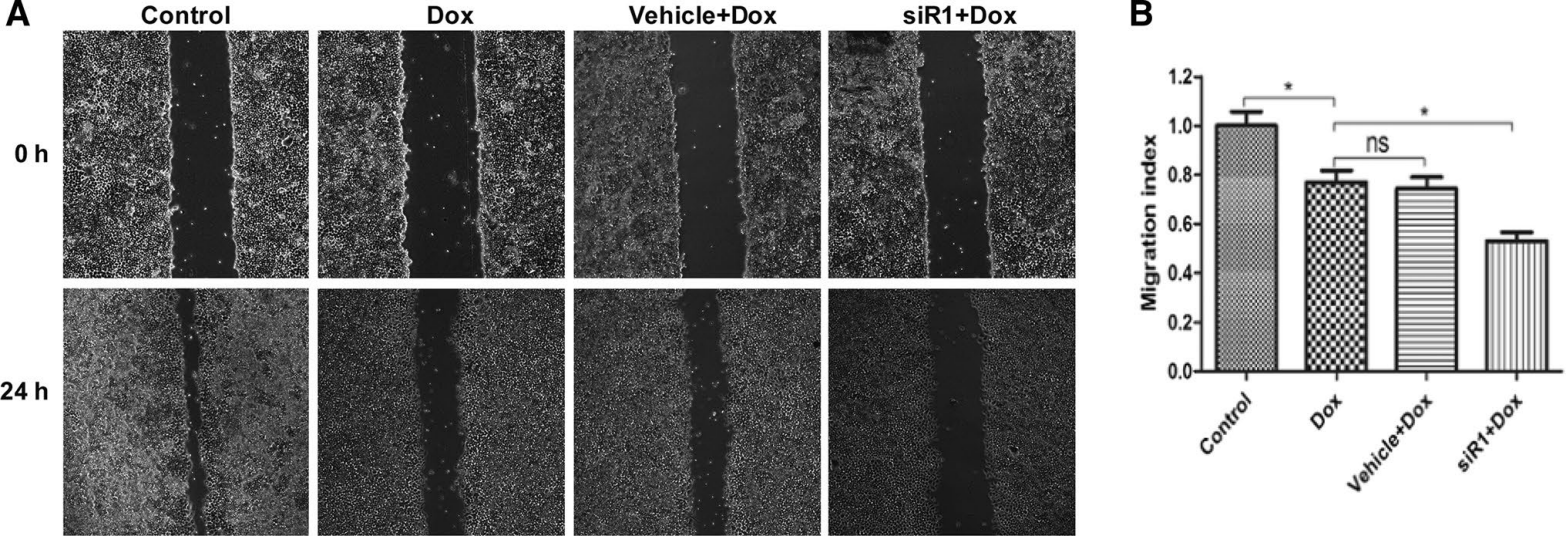
Scratch wounding assay for analysis of cell migration. **a** Cell migration imagines at 0 h and 24 h with CIP2A siRNA and doxorubicin administration, **b** migration index of MCF-7/ADR cells *P < 0.05. *Dox* doxorubicin, *siR1* CIP2A RNA interference 1, *NS* no significant

In wound healing assay, we observed that MCF-7/ADR cells migration capacity was inhibited by doxorubicin (40 μM). Moreover, cell migration index was further decreased to a greater extent in siR1 plus doxorubicin-treated cells compared to doxorubicin treatment alone. (Fig. 3a, b).

**Fig. 4:**
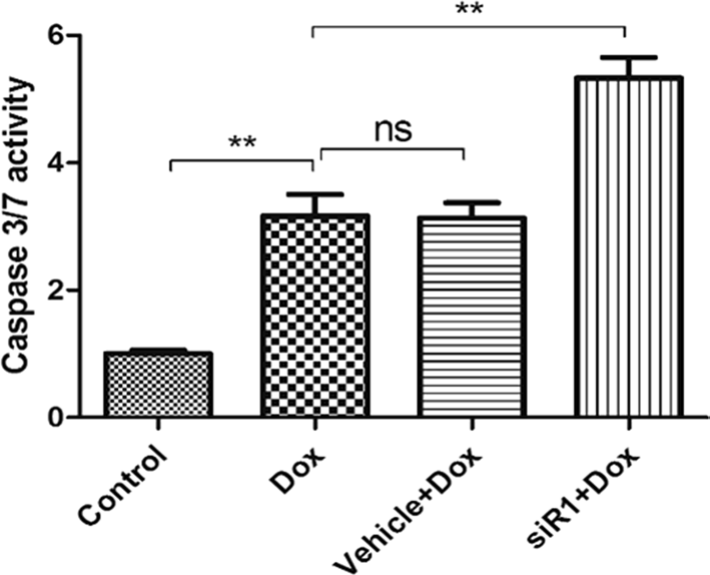
Effect of CIP2A silencing on apoptosis. Caspase 3/7 activity of MCF-7/ADR cells with treatment of doxorubicin. **P < 0.01 compared to control

To explore the impacts of *CIP2A* silencing on cell apoptosis, we measured caspase-3/7 activity and found that knocking down *CIP2A* further promoted apoptosis even in the presence of doxorubicin (Fig. 4).

### CIP2A silencing upregulates PP2A activity and cell autophagy

**Fig. 5:**
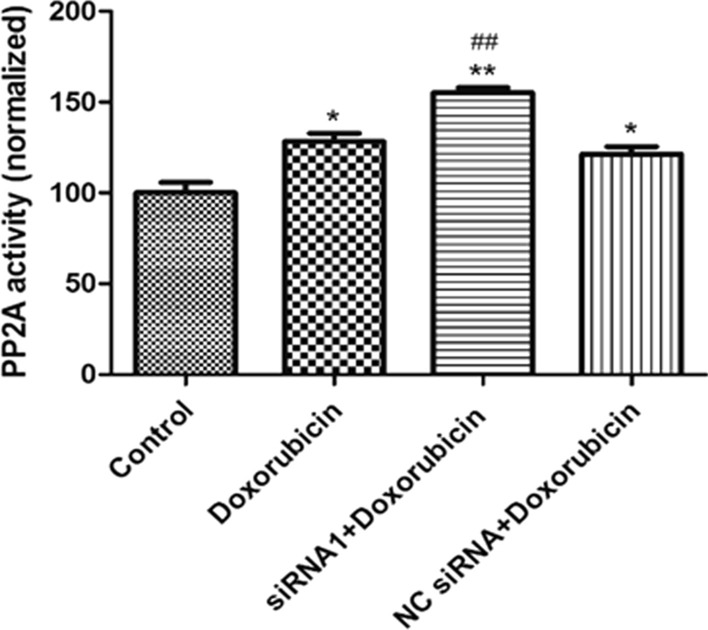
Effect of CIP2A silencing on PP2A activity. *P < 0.05, **P < 0.01 compared to control, ^##^*P* < 0.01 compared to doxorubicin only group

CIP2A was previously recognized as a molecular inactivator of PP2A [18]. Here, we analyzed the PP2A activity in MCF-7/ADR cells after doxorubicin treatment and silencing *CIP2A*. Our result indicted that PP2A activity was induced by doxorubicin alone, while knocking down *CIP2A* could further enhance PP2A activity to a greater level, even in the presence of doxorubicin (Fig. 5).

**Fig. 6:**
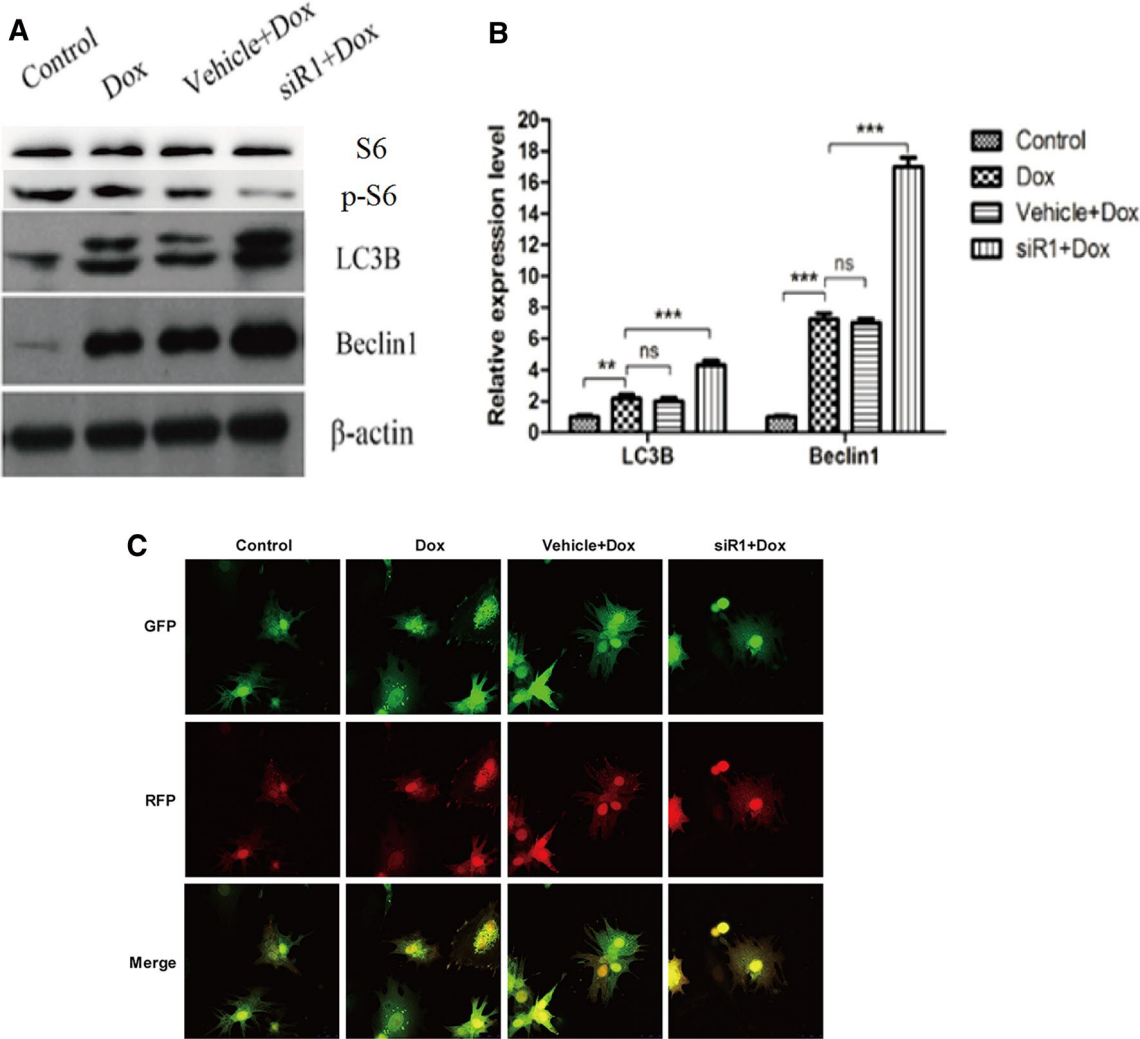
Expression of markers for autophagy. **a** Western blotting assay. **b** Quantification of the western blotting bands. **P < 0.01, ***P < 0.001. *Dox* doxorubicin, *NS* no significant. **c** Laser scanning confocal microscopy photos indicating autophagy flux

Previous literatures have suggested the involvement of CIP2A in promoting mTORC1-dependent cell growth and autophagy inhibition [17]. By analyzing the autophagy markers, we found that downregulation of *CIP2A* induced LC3B and Beclin1 at protein levels in MCF7/ADR cells (Fig. 6a, b). Additionally, mTOR downstream target p-S6 was also significantly decreased in the presence of siR1. More importantly, by transducing MCF7/ADR cells with mRFP-GFP-LC3 adenovirus, we have found the siR1 significantly promoted autophagy flux indicated by increased yellow and red puncta (Fig. 6c).

## Discussion

Cancer cells possess innate or acquired abilities to evade from chemotherapies, which is a major obstacle for the development of anti-cancer treatments. The mechanisms of chemoresistance include altered metabolism of glutathione [19], reduced drug accumulation resulting from enhanced efflux, differential oxygen free-radical susceptibility [20], increased DNA damage repair and genetic alterations [21]. During doxorubicin treatment, the drug resistance occurs and accompanied with unique morphological changes [22]. Resistance to doxorubicin represents a key issue towards successful treatment of breast cancer. Here, our study suggested that CIP2A inhibition could promote cell death even in cancer cells that are resistant to doxorubicin.

The doxorubicin-resistant cell line could be obtained with doxorubicin treatment at 200 nmol/L and culture for 1 month [23]. To determine the concentration at which doxorubicin could elicit an effective anti-proliferative response, we found that 40 μM doxorubicin caused a robust inhibition of proliferation in MCF-7/ADR cells. Similar resistance effect against doxorubicin has been reported using MDA-MB cell line [23]. High level of the oncogene CIP2A occurs frequently in many cancer types and correlates with malignant growth and clinical aggressiveness. Hence, CIP2A is considered as a predictive marker of cancer prognosis [24]. Other study suggested that CIP2A may play a role in anti-cancer growth [25]. However, the mechanism of CIP2A in the modulation of drug resistance remains undefined. In current study, *CIP2A* silencing leads to decreased cell viability in MCF-7 cells in addition to doxorubicin-mediated effects, suggesting that targeting CIP2A may represent a potential therapeutic strategy to treat breast cancer patients who developed resistance to doxorubicin. Constantly, previous reports also suggested that increased CIP2A expression may confer to doxorubicin resistance [15].

Previous study found that knocking down *CIP2A* expression inhibited cell proliferation of human multiple myeloma cells and induced early apoptosis via inactivation of PI3K/AKT/mTOR signaling [26]. *CIP2A* was also identified as a critical molecule that contributes to bortezomib-induced apoptosis via regulation of AKT signaling pathway [27]. Our results revealed that *CIP2A* siRNA raised caspase-3/7 level and promoted death. In addition, significant migration inhibition measured by scratch assay was also observed in the *CIP2A* siRNA-transfected cells. Moreover, the expression level of CIP2A is quite low in healthy human tissues [6], therefore targeting *CIP2A* may represent a potential therapeutic strategy for anti-breast cancer therapies.

There are also reports showing that CIP2A regulates cancer cell behavior through inhibiting PP2A activity [25]. Additionally, the activity of PP2A is considered as a prognostic marker for doxorubicin resistance and the outcome of breast cancer patients [28]. We found that *CIP2A* silencing upregulated PP2A activity significantly. These observations support the potential benefits of CIP2A inhibition for breast cancer treatment via activating PP2A.

In current study, we found that downregulation of CIP2A elevated the expression levels of LC3B and Beclin1 of MCF-7/ADR cells. This result indicated that autophagy activity was enhanced by silencing *CIP2A*. Many studies had pointed that CIP2A is involved in the regulation of the mTORC1 and autophagy [17, 24, 29]. CIP2A is not only proven to be able to inhibit autophagy but also its degradation can be elevated by autophagy activation [17, 30]. Blockade of PP2A activity inhibits autophagy by activating mTOR pathway [31], which is in line with our results that knocking down *CIP2A* upregulates PP2A and promotes autophagy. Moreover, given the fact that CIP2A inhibitors are commercially available, CIP2A represents a better target for breast cancer intervention compared to PP2A, because the latter has a wide range of phosphorylation function for multiple substrates and might give rise to various unnecessary side effects.

## Conclusion

Our data provide direct evidence that silencing *CIP2A* suppressed the proliferation of doxorubicin-resistant MCF-7 cells, induced apoptotic activity, inhibited cell migration significantly and promoted autophagy. These CIP2A-mediated effects are at least partially due to the upregulation of PP2A activity. The underlying mechanism by which *CIP2A* regulates PP2A activity and other cellular functions could to be explored in future studies, which will further advance our knowledge in terms of the function of CIP2A and facilitates the finding of new targets for anti-cancer drug development.

## Supplementary Information

Below is the link to the electronic supplementary material.

Supplementary file1 (TIF 82 KB)

## References

[1] Rangarajan A, Hong SJ, Gifford A, Weinberg RA (2004). Species—and cell type-specific requirements for cellular transformation. Cancer Cell.

[2] Zhao JJ, Roberts TM, Hahn WC (2004). Functional genetics and experimental models of human cancer. Trends Mol Med.

[3] Eichhorn PJ, Creyghton MP, Bernards R (2009). Protein phosphatase 2A regulatory subunits and cancer. Biochim Biophys Acta.

[4] Zhang Y, Fang L, Zang Y, Ren J, Xu Z (2018). CIP2A promotes proliferation, invasion and chemoresistance to cisplatin in renal cell carcinoma. J Cancer.

[5] Routila J, Makela JA, Luukkaa H, Leivo I, Irjala H, Westermarck J, Makitie A, Ventela S (2016). Potential role for inhibition of protein phosphatase 2A tumor suppressor in salivary gland malignancies. Genes Chromosomes Cancer.

[6] Junttila MR, Puustinen P, Niemela M, Ahola R, Arnold H, Bottzauw T, Ala-aho R, Nielsen C, Ivaska J, Taya Y (2007). CIP2A inhibits PP2A in human malignancies. Cell.

[7] Ma L, Wen ZS, Liu Z, Hu Z, Ma J, Chen XQ, Liu YQ, Pu JX, Xiao WL, Sun HD (2011). Overexpression and small molecule-triggered downregulation of CIP2A in lung cancer. PLoS ONE.

[8] Mathiasen DP, Egebjerg C, Andersen SH, Rafn B, Puustinen P, Khanna A, Daugaard M, Valo E, Tuomela S, Bottzauw T (2012). Identification of a c-Jun N-terminal kinase-2-dependent signal amplification cascade that regulates c-Myc levels in ras transformation. Oncogene.

[9] Come C, Laine A, Chanrion M, Edgren H, Mattila E, Liu X, Jonkers J, Ivaska J, Isola J, Darbon JM (2009). CIP2A is associated with human breast cancer aggressivity. Clin Cancer Res.

[10] Lucas CM, Harris RJ, Giannoudis A, Copland M, Slupsky JR, Clark RE (2011). Cancerous inhibitor of PP2A (CIP2A) at diagnosis of chronic myeloid leukemia is a critical determinant of disease progression. Blood.

[11] Chan S, Friedrichs K, Noel D, Pinter T, Van Belle S, Vorobiof D, Duarte R, Gil Gil M, Bodrogi I, Murray E (1999). Prospective randomized trial of docetaxel versus doxorubicin in patients with metastatic breast cancer. J Clin Oncol.

[12] Ganapathi RN, Ganapathi MK (2013). Mechanisms regulating resistance to inhibitors of topoisomerase II. Front Pharmacol.

[13] Taylor CW, Dalton WS, Parrish PR, Gleason MC, Bellamy WT, Thompson FH, Roe DJ, Trent JM (1991). Different mechanisms of decreased drug accumulation in doxorubicin and mitoxantrone resistant variants of the MCF7 human breast cancer cell line. Br J Cancer.

[14] Dunkern TR, Wedemeyer I, Baumgartner M, Fritz G, Kaina B (2003). Resistance of p53 knockout cells to doxorubicin is related to reduced formation of DNA strand breaks rather than impaired apoptotic signaling. DNA Repair (Amst).

[15] Choi YA, Park JS, Park MY, Oh KS, Lee MS, Lim JS, Kim KI, Kim KY, Kwon J, di Yoon Y (2011). Increase in CIP2A expression is associated with doxorubicin resistance. FEBS Lett.

[16] Liu X, Duan C, Ji J, Zhang T, Yuan X, Zhang Y, Ma W, Yang J, Yang L, Jiang Z (2017). Cucurbitacin B induces autophagy and apoptosis by suppressing CIP2A/PP2A/mTORC1 signaling axis in human cisplatin resistant gastric cancer cells. Oncol Rep.

[17] Puustinen P, Rytter A, Mortensen M, Kohonen P, Moreira JM, Jaattela M (2014). CIP2A oncoprotein controls cell growth and autophagy through mTORC1 activation. J Cell Biol.

[18] Lucas CM, Scott LJ, Carmell N, Holcroft AK, Hills RK, Burnett AK, Clark RE (2018). CIP2A- and SETBP1-mediated PP2A inhibition reveals AKT S473 phosphorylation to be a new biomarker in AML. Blood Adv.

[19] Batist G, Tulpule A, Sinha BK, Katki AG, Myers CE, Cowan KH (1986). Overexpression of a novel anionic glutathione transferase in multidrug-resistant human breast cancer cells. J Biol Chem.

[20] Mimnaugh EG, Dusre L, Atwell J, Myers CE (1989). Differential oxygen radical susceptibility of adriamycin-sensitive and -resistant MCF-7 human breast tumor cells. Cancer Res.

[21] Ridinger J, Koeneke E, Kolbinger FR, Koerholz K, Mahboobi S, Hellweg L, Gunkel N, Miller AK, Peterziel H, Schmezer P (2018). Dual role of HDAC10 in lysosomal exocytosis and DNA repair promotes neuroblastoma chemoresistance. Sci Rep.

[22] Yang JY, Ha SA, Yang YS, Kim JW (2010). p-Glycoprotein ABCB5 and YB-1 expression plays a role in increased heterogeneity of breast cancer cells: correlations with cell fusion and doxorubicin resistance. BMC Cancer.

[23] Smith L, Watson MB, O'Kane SL, Drew PJ, Lind MJ, Cawkwell L (2006). The analysis of doxorubicin resistance in human breast cancer cells using antibody microarrays. Mol Cancer Ther.

[24] Liu X, Cao W, Qin S, Zhang T, Zheng J, Dong Y, Ming P, Cheng Q, Lu Z, Guo Y (2017). Overexpression of CIP2A is associated with poor prognosis in multiple myeloma. Signal Transduct Target Ther.

[25] Niemela M, Kauko O, Sihto H, Mpindi JP, Nicorici D, Pernila P, Kallioniemi OP, Joensuu H, Hautaniemi S, Westermarck J (2012). CIP2A signature reveals the MYC dependency of CIP2A-regulated phenotypes and its clinical association with breast cancer subtypes. Oncogene.

[26] Yang X, Zhang Y, Liu H, Lin Z (2016). Cancerous inhibitor of PP2A silencing inhibits proliferation and promotes apoptosis in human multiple myeloma cells. Biomed Res Int.

[27] Tseng LM, Liu CY, Chang KC, Chu PY, Shiau CW, Chen KF (2012). CIP2A is a target of bortezomib in human triple negative breast cancer cells. Breast Cancer Res.

[28] Rincon R, Cristobal I, Zazo S, Arpi O, Menendez S, Manso R, Lluch A, Eroles P, Rovira A, Albanell J (2015). PP2A inhibition determines poor outcome and doxorubicin resistance in early breast cancer and its activation shows promising therapeutic effects. Oncotarget.

[29] Puustinen P, Jaattela M (2014). KIAA1524/CIP2A promotes cancer growth by coordinating the activities of MTORC1 and MYC. Autophagy.

[30] Chen KF, Liu CY, Lin YC, Yu HC, Liu TH, Hou DR, Chen PJ, Cheng AL (2010). CIP2A mediates effects of bortezomib on phospho-Akt and apoptosis in hepatocellular carcinoma cells. Oncogene.

[31] Magnaudeix A, Wilson CM, Page G, Bauvy C, Codogno P, Leveque P, Labrousse F, Corre-Delage M, Yardin C, Terro F (2013). PP2A blockade inhibits autophagy and causes intraneuronal accumulation of ubiquitinated proteins. Neurobiol Aging.

