# Supplementary figures and images for: CIP2A silencing alleviates doxorubicin resistance in MCF7/ADR cells through activating PP2A and autophagy
Source: Clin Transl Oncol. 2021 May 4;23(8):1542–8. doi: 10.1007/s12094-021-02616-7 (PMC8238779; doi:10.1007/s12094-021-02616-7)

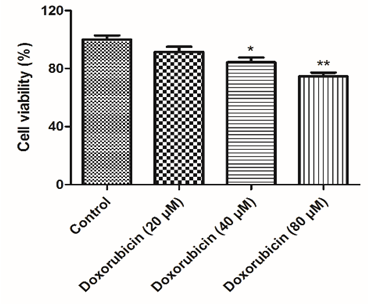

Supplement: Supplementary file 1 — Supplementary file1 (TIF 82 KB) [file 12094_2021_2616_MOESM1_ESM.tif]
